# Beyond COVID-19 Vaccination: Global Human Unity and Ensuing Economic Alliances

**DOI:** 10.3389/fpubh.2021.769764

**Published:** 2021-12-24

**Authors:** Rene O. Beleboni, Eduardo L. V. Silveira, Rodrigo G. Stabeli

**Affiliations:** ^1^Department of Biotechnology, School of Medicine, Ribeirão Preto University, Ribeirão Preto, Brazil; ^2^LABI (Laboratory of B Cell Immunology), Department of Clinical and Toxicological Analyses, School of Pharmaceutical Sciences, University of São Paulo, São Paulo, Brazil; ^3^Federal University of São Carlos (UFSCAR), São Carlos, Brazil; ^4^Departamento de Medicina, Plataforma de Medicina Translacional, Fiocruz-SP, Ribeirão Preto, UFSCAR e Unidade de Emergência, preparação e catástrofe em Saúde Pública, OPAS/OMS, Brasilia, Brazil; ^5^Fundação Oswaldo Cruz, Bi-Institutional Plaform of Translational Medicine, Oswaldo Cruz Foundation, Ribeirão Preto, Brazil

**Keywords:** COVID-19 pandemic, anti-vaccine movement, fake news, developing countries, economics

## A Need for Global Human Unity: Vaccines Save Lives

After a year and a half of the continuous 2019 coronavirus disease (COVID-19) pandemic, the world has witnessed a devastating effect on humanity. Due to its highly efficient transmission pattern, the severe acute respiratory syndrome coronavirus 2 (SARS-CoV-2) has rapidly collapsed public and private healthcare systems worldwide. Along with the long-term hospitalization, high mortality rate, and establishment of considerable sequelae among the convalescent patients, this virus has enormously contributed to the growing unemployment levels and the ensuing poverty in developing countries. To rescue their health systems and economies, it is imperative that these countries enhance their COVID-19 vaccine coverage as soon as possible.

Following the successful experiment conducted against smallpox performed by the English physician Edward Jenner in 1796, vaccines have been one of the most outstanding triumphs against infectious diseases. In particular, vaccines have significantly reduced human morbidity and mortality (1) alongside the usage of modern antibiotics, water sanitation, and technological advances in nutrition and hygiene/housing. These factors have been responsible for prolonging human life expectancy by an additional 30–40 years in different regions, particularly in the last eight decades and in most developed countries (2). Therefore, vaccines save lives.

To date, vaccines presumably save 4–5 million lives every year (3). This achievement became possible after the establishment of the WHO Expanded Programme on Immunization in 1977. Six of the most prevalent and preventable infectious diseases at that time began to be dealt with by vaccines. These diseases were tuberculosis, measles, poliomyelitis, whooping cough, tetanus, and diphtheria. As a part of a global initiative, this program has been continuously updated throughout the decades. It incremented critical inputs on vaccination success under different designations. For instance, smallpox has been successfully eradicated from the world, measles deaths have been reduced by almost 80%, tetanus has vanished from one-third of the locations containing positive cases, and the polio infection is about to be eliminated, except in Pakistan and Afghanistan where the respective national immunization programs have been disturbed by turbulent political and religious restrictions (4).

Several attempts have been made to set up drug repositioning against the SARS-CoV-2 and COVID-19. However, obtaining an effective and safe treatment for viral infections is very difficult, especially amid a pandemic. Hence, vaccination is the only option available for tackling COVID-19 so far.

## Anti-Vaccine Movement

The reputation and legitimacy of natural sciences have been put at risk due to some trending topics, vaccines being one of the most common targets. Several anti-vaccine movements have historically utilized ambiguous and suspect strategies to dismantle the primary message of vaccination campaigns: vaccines save lives. This conceptual threat is not novel, but with the onset and continuity of the COVID-19 pandemic, this tactic has gained strength with the support of some political leaders and ideological parties. From time to time, deliberate misinterpretations of reality remerge, and great efforts are required to avoid the discrediting of scientific conquests and the suffocation of the scientific community. Although this ongoing COVID-19 pandemic has caused more than 5 million fatalities worldwide in almost 2 years (5), the arguments encircling anti-vaccine groups persistently follow puzzling questions for veracity and social discussions surrounding human beings. More precisely, anti-vaccine groups have shown chaotic and confused interpretations of the scientific data related to vaccines (6).

In addition, there is a disseminated low public tolerance for vaccine-derived side effects, even being so far fewer and rarer than those elicited by the administration of conventional drugs (7–10). Rumors related to this issue have been historically propagated, creating an unnecessary disturbance in the vaccination coverage against different diseases worldwide. Network models have shown that the spread of fake news related to vaccines can result in harmful consequences for the population's adherence to the proper vaccination regimen when necessary (11).

These rumors and the uneven educational and economic levels among nations could have contributed to the unvaccination and ensuing deaths of millions of children of vaccine-preventable infections, such as pneumonia, every year. In 2019, a total of 13.6 million children did not receive any vaccine dose against diphtheria, tetanus, pertussis (DTP3), and measles, while 5.4 million dropped immunization out according to UNICEF and WHO official reports (12, 13). In the following year, the advent of the COVID-19 pandemic led to another considerable decline in the DTP3 vaccination, elevating concerns with the resurgence of vaccine-preventable diseases worldwide. More specifically, 17.1 million children did not receive a single DTP3 and measles vaccine dose, while 5.6 million dropped vaccination out. These 2020 numbers of children unvaccinated against DTP3 and measles were the highest since 2009 (13). On the other hand, one can assume that the inclusion of anti-COVID-19 formulations into the current vaccine portfolio will increase the number of lives saved by vaccination, overcoming the known estimates.

In the meantime, health professionals and scientists have undertaken tremendous efforts to solve the misinterpretation of data spread by fake news. It has been concluded that it is imperative to reinforce the following: (i) the continuous orientation of the population about verified COVID-19 facts (the number of cases, respective hospitalizations, and deaths as well as the vaccination progress) and the disease's potential for dissemination or recrudescence; (ii) the adoption of health security measures and hygienic conducts; (iii) trust in vaccination campaigns to enhance the population adhesion; (iv) a robust pharmacovigilance system in place to track down the manner in which mass vaccination progresses daily. The national immunization programs, national and international regulatory authorities for medicines, vaccine manufacturers, global agencies (i.e., WHO and UNICEF), and governments must facilitate a faster and more effective distribution of vaccine supplies and doses across the globe. Also, they can aid in the prevention and management of a potential crisis involving vaccination programs.

A continuous investment in science and technology pays off exactly when rapid actions are needed, such as the development of a vaccine against a pandemic. Since the early 2000s, several global pharmaceutical companies have increased their budgets for translational research. Resultant information applied to the current knowledge of genomics, proteomics, microbiology, nanotechnology, and immunology has improved essential aspects of vaccination, such as the reduction of cost and time for vaccine manufacturing, storage, safety, and efficacy (14). Based on the well-established and traditional scaffold of inactivated pathogens as preventive strategies, novel vaccine formulations have been subsequently proposed. Astonishingly, non-replicant viral vector-based and messenger RNA (mRNA) vaccines specific to the SARS-CoV-2 virus have been made, distributed, and even administered within a year of the emergence of the COVID-19 pandemic. This accelerated process of vaccine manufacturing can be explained by the massive efforts undertaken by the scientific and industrial communities that have been evaluating these options as potential formulations against other pathogens for a long time. Considering the sense of urgency globally created by COVID-19, time and funds have been substantially invested in vaccine development. Governments all around the world have spent almost 100 billion euros for developing COVID-19 vaccines (15). Hence, the current analysis of technical and regulatory information facilitates the process of making vaccines, thereby allowing a quick evaluation of vaccine safety and efficacy. Countries with the highest numbers of COVID-19 cases and deaths could have had their population enrolled in large vaccine clinical trials. Furthermore, the unprecedented parallel that facilitated the real-time conversation between regulatory agencies for medicines and vaccine manufacturers made the process of vaccine licensing quicker during this pandemic. However, these actions have not resulted in a massive vaccine coverage in developing countries yet as expected.

Since the licensing of the first anti-SARS-CoV-2 vaccine prototypes, a relentless race has commenced worldwide to purchase these immunobiologicals. Owing to their higher economic structure and political and scientific/industrial organization, the most powerful countries have produced, and/or obtained the majority of the available vaccine doses. Some of these countries have acquired more vaccine doses than the size of their respective populations. In contrast, many developing countries have struggled with fake news, science negationism, and political resistance while acquiring vaccines and/or concluding negotiations. Given the changes in patent rules and reorganization of the pharmaceutical and foreign trade scenarios in the last 40 years, these countries have lagged in technological development as compared to their developed counterparts. Hence, developing countries have created an enormous dependence on the supply of pharmaceutical inputs from abroad. In a situation where these places lost pharmaceutical infrastructure for research, development, and production even for basic medicines, their political, social, and economic actions showed no regrets concerning these issues (16).

At present, many developing countries are desperately begging for vaccines as the rates of hospitalization and death among their patients with COVID-19 are skyrocketing. In contrast, their productivity has declined in multiple areas, reducing their gross domestic product (GDP) as previously estimated (17). These massive economic losses in internal and external business networks have disseminated poverty as a vicious cycle in those countries. Paradoxically, multiple countries with opposing strategies for facing the pandemic have started putting developing countries on the side. For instance, the fear of COVID-19 led them to refuse flights to or from developing countries where the viral dissemination was unleashed. As a consequence, cases of discrimination against people derived from developing countries have been horrendous reported (18–22).

Considering COVID-19 pandemic as a real public health issue overseas and the current interdependence among countries, it is evident that the efforts to fight the pandemic must also be part of a global strategy consisting of mutual assistance and humanistic unity. If the vaccination coverage does not proportionally increase worldwide, the new SARS-CoV-2 viral strains (including those potentially more transmissible and/or lethal) may continue to rise (23). The expansion of this scenario would abrogate all the global efforts and investments implemented to stop the pandemic, favoring the dissemination of the disease or recrudescence and hindering the rebirth of the global economy and politics carried out so far. Thus, the International Monetary Fund (IMF) has considered that reaching a 70% vaccination coverage worldwide in mid-2022 may stop these detrimental effects of the pandemic. As consequence, positive projections for the global economy have been made. While advanced economies would increase 5.2% in 2021, emerging markets and developing economies are expected to reach 6.4% at the same period. However, rising inflation can hold up this growth in 2022. To avoid this scenario, it is critical that pandemic-affected countries keep up with essential spending and trade issues set up before the pandemic be solved by the international community (24).

In order to return economic and social activities to full-gear intensity, developing countries must have access to more vaccine options and doses. This outline alone will allow them to speed up their vaccination rates against COVID-19. While corroborating this idea, some of the wealthiest countries have recently proposed ideas on managing this problem. For instance, the G7 countries have just announced a plan to donate one billion COVID-19 vaccine doses, which has not been cordially greeted by the United Nations (25). In addition, the American government signaled the possibility of waiving intellectual property on COVID-19 vaccines in contrast to the interests of the pharmaceutical companies that hold patents (26). Furthermore, the IMF has suggested to tackle postpandemic economy issues through human capital accumulation and usage of green technology and digitalization, which could diminish inequality and maintain the stability of public finances (24).

## Beyond COVID-19 Vaccination

Considering the urgency brought by this human tragedy, international solidarity is more than welcome for most developing countries. Poverty and infectious disease outbreaks, such as cholera, meningitis, avian influenza, and Ebola, in multiple East African communities have motivated several non-profit organizations followed by some of the G7-country governments in providing water, food, medicines, vaccines, and medical care. Besides these humanitarian activities, potential infectious diseases have been successfully inhibited from spreading out. Thus, we reiterate the importance of the wealthiest countries in commanding the combat of the COVID-19 pandemic worldwide and urge developing countries to continue investing in science, industry, innovation, education, and health. Moreover, as COVID-19 is not likely to be the last pandemic in the world, science and industrial infrastructure are as critical as the international aids potentially offered. The control or potential eradication of the COVID-19 pandemic through vaccination would reinforce the importance of science and industry as pillars for human society to battle any future epidemics. Furthermore, this current pandemic represents a valuable opportunity to avoid using political and ideological arguments against medical statements; to maintain or extend economic bonds; more importantly, to propagate humanitarianism and global union for ensuring humanity's survival.

## Author Contributions

RB: conceptualised and directed the project, searched for articles in the literature, prepared and reviewed the manuscript. ES: conceptualised and directed the project, searched for articles in the literature, prepared and reviewed the manuscript. RS: conceptualised and directed the project, searched for articles in the literature, prepared and reviewed the manuscript. All authors contributed to the article and approved the submitted version.

## Funding

This work was supported by the Research productivity fellowship CNPq process - 309874/2017-3 to RB, Integrated Research Projects in Strategic Areas (PIPAE-USP) - Year 2021 - Edital 2021.1.10424.1.9 to ES.

## Conflict of Interest

The authors declare that the research was conducted in the absence of any commercial or financial relationships that could be construed as a potential conflict of interest.

## Publisher's Note

All claims expressed in this article are solely those of the authors and do not necessarily represent those of their affiliated organizations, or those of the publisher, the editors and the reviewers. Any product that may be evaluated in this article, or claim that may be made by its manufacturer, is not guaranteed or endorsed by the publisher.
